# An interprofessional nurse-led mental health promotion intervention for older home care clients with depressive symptoms

**DOI:** 10.1186/1471-2318-14-62

**Published:** 2014-05-10

**Authors:** Maureen Markle-Reid, Carrie McAiney, Dorothy Forbes, Lehana Thabane, Maggie Gibson, Gina Browne, Jeffrey S Hoch, Thomas Peirce, Barbara Busing

**Affiliations:** 1 School of Nursing, McMaster University, Hamilton, Ontario, Canada; 2 Department of Clinical Epidemiology and Biostatistics, McMaster University, Hamilton, Ontario, Canada; 3 Department of Psychiatry and Behavioural Neurosciences, McMaster University, Hamilton, Ontario, Canada; 4 St. Joseph’s Healthcare, Hamilton, Ontario, Canada; 5 Faculty of Nursing, University of Alberta, Edmonton, Alberta, Canada; 6 Veterans Care Program, Parkwood Hospital, St. Joseph’s Healthcare, London, Ontario, Canada; 7 Department of Health Policy, Management and Evaluation, University of Toronto, Toronto, Ontario, Canada; 8 Hamilton Niagara Haldimand Brant Community Care Access Centre, Brantford, Ontario, Canada

**Keywords:** Nurse-led interventions, Home care, Interdisciplinary, Depression management, Older adults, Clinical effectiveness

## Abstract

**Background:**

Depressive symptoms in older home care clients are common but poorly recognized and treated, resulting in adverse health outcomes, premature institutionalization, and costly use of health services. The objectives of this study were to examine the feasibility and acceptability of a new six-month interprofessional (IP) nurse-led mental health promotion intervention, and to explore its effects on reducing depressive symptoms in older home care clients (≥ 70 years) using personal support services.

**Methods:**

A prospective one-group pre-test/post-test study design was used. The intervention was a six-month evidence-based depression care management strategy led by a registered nurse that used an IP approach. Of 142 eligible consenting participants, 98 (69%) completed the six-month and 87 (61%) completed the one-year follow-up. Outcomes included depressive symptoms, anxiety, health-related quality of life (HRQoL), and the costs of use of all types of health services at baseline and six-month and one-year follow-up. An interpretive descriptive design was used to explore clients’, nurses’, and personal support workers’ perceptions about the intervention’s appropriateness, benefits, and barriers and facilitators to implementation.

**Results:**

Of the 142 participants, 56% had clinically significant depressive symptoms, with 38% having moderate to severe symptoms. The intervention was feasible and acceptable to older home care clients with depressive symptoms. It was effective in reducing depressive symptoms and improving HRQoL at six-month follow-up, with small additional improvements six months after the intervention. The intervention also reduced anxiety at one year follow-up. Significant reductions were observed in the use of hospitalization, ambulance services, and emergency room visits over the study period.

**Conclusions:**

Our findings provide initial evidence for the feasibility, acceptability, and sustained effects of the nurse-led mental health promotion intervention in improving client outcomes, reducing use of expensive health services, and improving clinical practice behaviours of home care providers. Future research should evaluate its efficacy using a randomized clinical trial design, in different settings, with an adequate sample of older home care recipients with depressive symptoms.

**Trial registration:**

Clinicaltrials.gov identifier: NCT01407926.

## Background

Depression affects 26-44% of older adults using home care services, in whom it is more prevalent and severe than in older persons in general [1-8]. Although late-life depression can be successfully treated with antidepressant medications or psychosocial interventions, few older home care clients receive adequate trials of such treatment or use specialized mental health services [1,8-12]. Untreated or under-treated depression in older adults is a serious public health problem [12,13], associated with greater morbidity and dependency, functional decline, diminished health-related quality of life (HRQoL), pain [14], poor adherence to medical treatment [15], increased demands on family caregivers, premature nursing home admissions [16], increased use of healthcare services [2,3,9,17-19], and increased risk of premature death from suicide and other medical conditions [20].

Older home care clients using personal support services (PSS), which are provided by personal support workers (PSWs), are at particularly high risk for depression, compared to other home care clients. These clients, who represent 75-80% of home care users [21], are typically over 70 years of age and have multiple health conditions [22], functional disabilities, cognitive impairment, or low social support [23-25]. These conditions are both risk factors for and outcomes of depression in older adults [2,26,27].

Many challenges to the diagnosis and management of depression in older adults have been identified, including difficulties disentangling coexisting medical, psychiatric, and social morbidity [9]; transportation and access difficulties; social isolation [28]; healthcare provider attitudes toward mental health disorders and treatment; and reluctance of older adults to accept the diagnosis of depression [3,29]. Thus, older home care recipients may be particularly vulnerable to suboptimal depression care and its negative outcomes. The magnitude of the problem has the potential to increase, because of the rising number of seniors [30], and the associated increase in the prevalence of depressive symptoms [26]. However, little is known about the prevalence of depression among community-living older adults using PSS [25].

Depression generally results from an interaction of multiple risk factors, many of which are modifiable, such as anxiety; persistent sleep difficulties; chronic stress associated with declining health, or family or marital problems; and social isolation [13,26]. Attention to these risk factors can reduce the prevalence and severity of depression [31]. The assessment of depressive symptoms in older adults has become a topic of concern for both clinicians and researchers, as evidenced by the emergence of many depression assessment tools for older adults [32-34]. Intervention studies have shown that, once identified, 80-90% of depressive disorders can be successfully treated [35,36].

Given the complex and multifactorial nature of depression, multicomponent, collaborative interventions provided by an interprofessional (IP) team [37,38], that involve use of standardized screening tools and antidepressant medications, psychotherapy [39-44], or psychosocial interventions tailored to individual needs [37] and preferences [45], have the greatest effect on reducing depression in this population. The benefit is even greater if the program targets individuals at risk of, suffering from, or recovering from depression [46]; incorporates clinician education [26,45,47]; and involves an enhanced role for the nurse [45,48], proactive follow-up and outcomes monitored by a care manager, enhanced IP communication, and integration between primary and specialist mental healthcare services [48-50]. These conclusions have been substantiated by systematic reviews and meta-analyses of many randomized controlled trials (RCTs) [32,45,51-53] and by consensus panels of experts who have developed evidence-based practice guidelines for the prevention, detection, and management of depression [9,26,54-56].

Many challenges exist to integrating these evidence-based strategies into practice in the home care setting. Considerable reorganization of the delivery of services to older adults with chronic needs may be required. However, home care is already underfunded. Home care clients at risk of, suffering from, or recovering from depression have limited access to professional services promoting mental health, especially nursing [57,58].

Other barriers to optimal depression care include inadequate collaboration and communication among home and community care providers, primary healthcare providers, and specialized mental healthcare providers; no continuity among providers; difficulties accessing specialized mental healthcare services; lack of knowledge among home care providers in recognizing and managing depression [28,58-61]; and underuse of depression screening tools. A final barrier is the lack of evidence-based practice standards specific to the assessment and management of depression in home care for older adults [58]. While routine screening for depressive symptoms among long-stay home care clients is conducted in Canada using the RAI-HC (http://www.interrai.org/home-care.html), its effectiveness is undetermined. Innovative strategies are needed to address these barriers and reduce the research-practice gap to improve the prevention, recognition, and management of depression in this underserved, high-risk population [62].

Models of care that facilitate IP collaborative practice are increasingly recognized as a means of addressing these barriers and improving client outcomes, because they lead to more efficient and effective use of healthcare resources and healthcare providers’ skills [63]. Community nurses are in an ideal position to lead an IP mental health promotion intervention for depression, given their scope of practice [64,65]. RCTs support the effectiveness of collaborative IP registered nurse (RN)-led, depression care management (DCM) interventions among older adults with depressive symptoms in primary care [40-42,66-83], home care [84,85], and institutional settings [86,87]. Nurse-led DCM typically involves a trained nurse care manager who works in collaboration with primary care providers, specialized mental healthcare providers, and other members of the IP team, to provide depression screening, outreach, and treatments [88,89].

Relatively little is known, however, about the effectiveness of IP nurse-led DCM interventions among older home care clients with depressive symptoms who are using PSS. To date, most RCTs of nurse-led DCM interventions for depressed older adults have been based in institutional or primary care settings [34]. In addition, little is known about the factors that mediate the changes in depressive symptoms brought about by these DCM interventions.

Studies based in the home care setting have shown that nurse-led DCM interventions improved detection of depression [3,61,85,90], reduced depressive symptoms [85,91,92], and lowered hospitalization rates [93-95], but they involved only short-term screening and referral for depression, used weaker study designs, had small sample sizes, or excluded older adults with dementia [28,34,56]. However, there is promising evidence from our previous RCT, which showed that a six-month nursing health promotion intervention, directed toward a general population of older home care clients using PSS provided by PSWs, compared with providing nursing services on demand, resulted in increased mental health functioning and related quality of life and a reduction in the severity of depressive symptoms, at no additional cost [5]. Further, there is evidence from two clinical trials of the effectiveness and feasibility of RNs working with PSWs in reducing depressive symptoms among older adults in long-term care settings [87,96].

The present study builds upon this work by testing a new IP mental health promotion intervention involving proactive follow-up by nurse (RN) care managers working collaboratively with the PSW, the home care case manager, the client’s primary care physician (PCP), and other IP home care providers (e.g., occupational therapy, physiotherapy, and social work), among older adults with depressive symptoms. The specific objectives of this study were to evaluate the feasibility and acceptability of the intervention and to explore its effects on reducing depressive symptoms in older home care clients with depressive symptoms using PSS. Our primary hypothesis was that an IP nurse-led mental health promotion intervention, delivered to older home care clients with depressive symptoms, would result in a reduction in the severity of these symptoms. Further, we hypothesized that the intervention would reduce anxiety, improve HRQoL, and pay for itself by reducing the use of expensive healthcare resources, such as hospitalization.

### Research questions

1. What is the feasibility and acceptability of the IP nurse-led mental health promotion intervention in the home care setting?

2. Does a six-month IP nurse-led mental health promotion intervention reduce depressive symptoms and anxiety, and improve HRQoL?

3. Does the intervention improve home care provider outcomes (depression management knowledge and practices)?

4. Does the intervention reduce the six-month and one-year costs of use of health services, from a societal perspective?

## Methods

This study was conducted in accordance with the Tri-Council Policy Statement, *Ethical Conduct for Research Involving Humans*[97]. Ethics approval for the study was obtained from the McMaster University Research Ethics Board (#10-041) and was renewed yearly as required. All participants provided written informed consent.

### Study design

Due to the complexity of evaluating health services interventions, a multiple method design (QUANT + qual) was used to examine the interplay among the home care context, implementation of the nurse-led strategy, and outcomes [98]. A prospective one-group, pre-test/post-test study design was used to evaluate the effectiveness of the IP nurse-led mental health promotion intervention, implemented under real-world conditions that included reliance on usual care providers. Assessments were made at baseline (pre-test) and immediately following the six-month intervention period (post-test). We also assessed outcomes a third time - six months after the intervention period - to assess sustainability of the intervention effects, which has been recognized as a gap in the literature [34]. An interpretive descriptive design was used to explore clients’, nurses’, and PSWs’ perceptions about the intervention’s appropriateness, benefits, and barriers and facilitators to implementation.

### Participants and setting

This study was a collaborative project between researchers at McMaster University and the University of Alberta, and decision-makers and practitioners in the Hamilton Niagara Haldimand Brant (HNHB) Community Care Access Centre (CCAC), Ontario Ministry of Health and Long-Term Care, HNHB Local Health Integration Network, Canadian Coalition for Seniors’ Mental Health, Canadian Mental Health Association, and two direct care provider agencies (Care Partners and ParaMed Home Healthcare) in Ontario, Canada. The CCAC provides publicly funded home care using a contractual model of service delivery, wherein case managers contract out home care services to agencies that provide care to clients.

Study participants were long-stay (> 60 days) home care clients, 70 years or older, newly referred to and receiving PSS through the CCAC, living in the community (not in a long-term care home), mentally competent to give informed consent (or with a substitute decision-maker available), competent in English (or with an interpreter available), not receiving palliative care services, and identified as having depressive symptoms.

### Screening for eligibility and enrolment

Trained CCAC case managers (CMs) identified potential participants based on the inclusion criteria, then contacted them by telephone to screen for depressive symptoms using the PHQ-2 questionnaire. An older person was deemed to have depressive symptoms and thus be eligible for the study if he/she answered “yes” to either of the following questions: Over the last two weeks, a) have you lost interest or pleasure in doing things most of the day, more days than not? or b) have you felt down, depressed, or hopeless most of the day, more days than not? [99]. Individuals with newly detected depressive symptoms as well as those who were already receiving treatment for depressive symptoms were eligible.

A research assistant conducted an in-home interview to obtain written informed consent and complete the baseline questionnaires. Older adults were deemed to be mentally competent and thus eligible for the study if they scored ≥ 24 on the Standardized Mini-Mental State Examination (SMMSE) [100]. Those who scored < 24 could be included if they had a substitute decision-maker to provide consent and complete the questionnaires on their behalf. All eligible and consenting participants were assigned to the IP nurse-led intervention and given a pamphlet with general study information.

### Intervention

**Figure 1:**
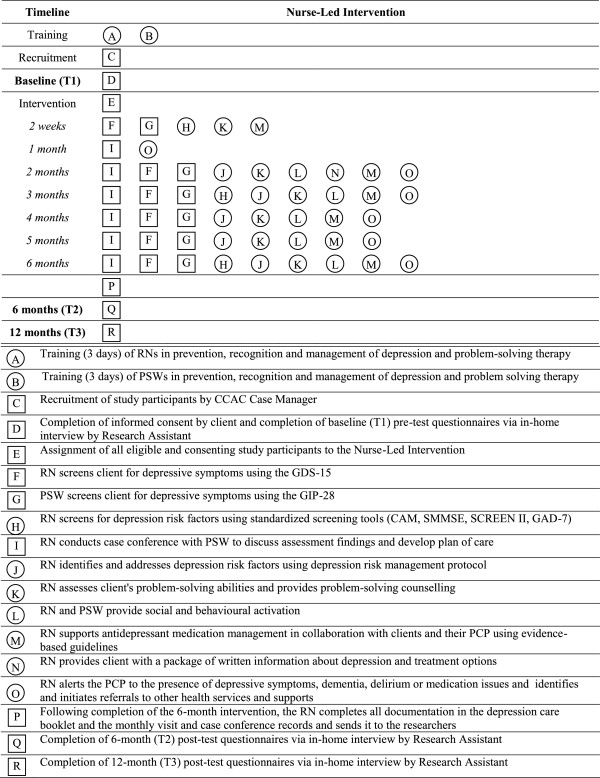
**Graphical depiction of intervention and measurements.** Squares represent fixed elements. Circles represent activities that are flexible. Measurements are bolded. This graphical method was proposed by Perera et al.

A detailed description of the intervention can be found elsewhere [101]. The intervention was a multifaceted six-month strategy led by an RN that was designed to detect, manage, and reduce the severity of depressive symptoms among older home care clients. The intervention was provided by a designated team of RNs and PSWs from two different direct care provider agencies. The RN and PSW worked collaboratively within an interprofessional team consisting of the Home Care Case Manager, the Primary Care Physician, and other home care providers. The RN provided intensive case management and community navigation to facilitate access to services and supports across the care continuum, provided psychosocial support and advocacy, and coordinated communication among the client, their family caregiver, and the IP team. Figure 1 summarizes the steps of the intervention.

Each participant was offered a monthly in-home visit by the RN and PSW for six months, in addition to usual home care services. The RN and PSW team’s main activities during the home visits, which lasted about one hour, included: (1) conducting comprehensive screening for depressive symptoms and risk factors for depression using validated tools; (2) assessment of depressive symptoms using the Geriatric Depression Scale (GDS)-15 [102] and the behavioural rating scale for intramural psychogeriatric inpatients (GIP-28) [103], and modification of risk factors for depression using the depression risk management protocol; (3) conducting a medication review and supporting antidepressant medication management in collaboration with clients and their PCP using best practice guidelines [26,27]; (4) educating the client and family caregiver about depression using printed educational materials; (5) assessing the client’s problem-solving strengths and limitations using the problem-solving test and providing problem-solving therapy (PST) using Nezu et al.’s [104] manual; (6) providing social and behavioural activation, which involved assisting and encouraging clients to participate in a regular physical activity program tailored to individual needs; (7) providing intensive support to both the client and their family caregiver; and (8) integrating depression care with ongoing care for other chronic conditions. The RN and PSW team tailored their visits to individual client needs.

Each participant’s treatment regimen was discussed by each RN and PSW dyad at a case conference held a minimum of once per month for six months. A depression care booklet was used to systematically guide the RN and PSW team through a series of questions that triggered assessment of depressive symptoms, current treatment, treatment response, risk factors for depression, use of social and behavioural activation, and PST, and recommended actions for reducing depressive symptoms and improving HRQoL. During the case conference, the RN and PSW team developed an evidence-based and client-centred depression management plan. The plan included specific short-term and six-month goals, a list of actions and referrals, a record of all recommendations, and the client’s response to treatment. The RN worked collaboratively with the Home Care Case Manager, the client’s PCP and other providers to initiate referrals to a comprehensive range of services and supports to address individual client needs, e.g., specialized mental health services. The RN also alerted the PCP to the presence of depression, dementia, or delirium using a standard letter, requesting further assessment and treatment. Table 1 provides an overview of the role of the RN versus the PSW in the delivery of the nurse-led intervention. Table 2 provides a summary of the key features of the nurse-led intervention compared to usual home care services.

**Table 1:** Role of the registered nurse versus the personal support worker in the interprofessional nurse-led intervention

**Registered nurse**	**Personal support worker**
Provides care coordination and liaises with interprofessional (IP) team, including the home care case manager, the primary care physician, and other home care providers	Monthly case conferences with the RN to discuss client status and response to treatment
In-home assessment for depressive symptoms using the GDS-15	In-home assessment for depressive symptoms using the GIP-28
In-home assessment for depression risk factors using standardized tools	Identifies and reports depression risk factors to the RN
Conducts medication review and supports antidepressant management in collaboration with clients and their primary care physician using best practice guidelines.	Reminds clients about medications, monitors medication use, monitors and reports side effects, worsening of symptoms, suicide ideation and risk and other behaviors to the RN, assists clients with medications as required
Provides social and behavioral activation	Provides social and behavioral activation and an in-home exercise program
Assesses the client’s problem-solving strengths and limitations using the problem-solving test and provides problem-solving therapy	Provides problem-solving therapy in collaboration with the RN
Provides client and caregiver with intensive support	Provides client and caregiver with intensive support
Educates the client and family caregiver about depression using printed materials	

**Table 2:** Interprofessional nurse-led mental health promotion intervention versus usual home care services

**Characteristics**	**IP Nurse-Led mental health promotion intervention**	**Usual home care**
***Home Care Service Providers***	Dedicated team of RNs and PSWs with specialized training in depression care for community-living older adults. RN and PSW dyad work collaboratively with the interprofessional (IP) team, which includes the Home Care Case Manager, the Primary Care Physician, and other home care providers in delivering the intervention.	No dedicated team of home care service providers with expertise in depression care for older adults
***Continuity of Care Provider***	Continuity of home care service provider through the use of a dedicated team of RNs and PSWs	Continuity of care provider not assured
***Depression Risk Factor Assessment Tools***	In-home assessment of depressive symptoms, and risk factors for depression using validated tools.	No standardized assessment tools across disciplines
***Depression screening, early identification, and management***	Implementation of evidence-based strategies for screening, early identification and management of depression. During the home visit, the RN and PSW dyad screens the client for depressive symptoms and depression risk factors, and provides problem-solving therapy, social and behavioural activation, medication review and antidepressant medication management, education about depression, and intensive support to both the client and their family caregiver	No evidence-based practice standard specific to the assessment and management of depression in home care for older adults
***Access to Home Care Services for Mental Health Promotion***	Monthly in-home visits by RN and PSW over six months for older adults with depressive symptoms.	Delayed or minimal access to professional home care services directed toward mental health promotion. Eligibility for home care services is based on physical/medical needs; not mental health needs
***Mechanisms for Team Communication and Collaboration***	Monthly case conferences involving the unique RN and PSW dyad assigned to each study participant	Limited communication and collaboration among team members and lack of inclusion of the PSW in the care team
***Information Systems***	A single evidence-based depression care management plan among members of the IP team	No formal mechanisms for shared record keeping across disciplines

A total of 13 home care providers (5 RNs and 8 PSWs) delivered the intervention. A four-pronged approach was used to implement the nurse-led intervention. First, the investigators held a one-day educational workshop with the RNs and PSWs together. Then, two-day *educational workshops* were held for the RNs and PSWs separately, supplemented by an eight-hour training workshop for the PSWs in the delivery of the Home Support Exercise Program [105]. Once the workshops were completed, the intervention was implemented using a multifaceted approach. An implementation team, consisting of the principal investigator, the research coordinator, and the managers from the participating care provider agencies, conducted monthly *outreach visits* with the intervention providers to discuss the progress of the study, provide feedback and education, and discuss barriers encountered and possible solutions. As *reminders*, the implementation team periodically provided updates on the study to their staff, including successes and areas for improvement. The RNs and PSWs were asked to record the intervention-specific activities that were carried out following each home visit and case conference [106,107]. The principal investigator conducted monthly audits of this documentation to assess fidelity to treatment. The results of this review were used as an *audit and feedback* strategy [108].

### Variables and measures

Independent interviewers assessed participants at baseline, immediately following the six-month intervention period, and again six months after the intervention had been completed through a structured in-home interview lasting about one hour. Three interviewers, all RNs with previous experience working in community-based settings, were trained in consent and data collection procedures; inter-rater reliability was good. Table 3 provides an overview of all outcome variables and measures.

**Table 3:** Variables and measures

	**Variables**	**Measures**	**Timing of data collection**
**Demographic and Depression-Related Characteristics**	Age, Gender, Medical Diagnoses, History of Depression, Culture, Informal supports, Education, Living arrangement, Income, Marital status, Use of prescription medications, Recent stressful life event, Alcohol use, Sleep difficulties, Chronic pain	Sociodemographic Questionnaire	T_1_
**Screen**	Depressive Symptoms	Patient Health Questionnaire-2 (PHQ-2)	T_1_
	Cognitive Status	Standardized Mini-Mental State Examination (SMMSE)	T_1_, T_2_ and T_3_
**Feasibility of the Intervention**	Eligibility Rate	Research Activity Log	T_1_
	Enrolment Rate	Research Activity Log	T_1_
	Dose of the Intervention	Monthly Visit and Case Conference Record	T_2_
	Fidelity to Treatment	Fidelity Scale	T_2_
	Attrition Rate	Research Activity Log	T_2_ and T_3_
	Comparison between Dropouts and Completers	Research Activity Log	T_1_, T_2_ and T_3_
**Acceptability of the Intervention**	Perceptions of Intervention by Study Participants	Semi-Structured Interviews	T_3_
	Perceptions of Intervention by Home Care Providers	Focus Group Interviews	6 and 8 months following initiation of the intervention
**Effects of the Intervention**	Depressive Symptoms	Centre for Epidemiological Studies in Depression Scale (CES-D)	T_1_, T_2_ and T_3_
	Anxiety	Generalized Anxiety Disorder Screener (GAD-7) Scale	T_1_, T_2_ and T_3_
	Health-Related Quality of Life	SF-12v2 Health Survey	T_1_, T_2_ and T_3_
**Home Care Provider Outcomes**	Depression Treatment: Prescription Antidepressant Medication Use, Use of Specialized Mental Health Services	Health and Social Services Utilization Inventory (HSSUI)	T_1_, T_2_ and T_3_
	Depression Management Knowledge	Home Care Provider Questionnaire	T_3_
**Costs of Use of Health Services, from a Societal Perspective**	Health Services Utilization, from a Societal Perspective	HSSUI	T_1_, T_2_ and T_3_

T_1_: Baseline; T_2_: 6 months after baseline measures; T_3_: 12 months after baseline measures.

### Participant characteristics

Demographic characteristics were assessed using standard questions at baseline. Participants were asked about history of diagnosed depression and if they experienced known risk factors for depression, including chronic pain, persistent sleep difficulties, a recent stressful life event, or excessive alcohol consumption.

### Feasibility of the intervention

Feasibility of the intervention relates to the degree to which the participants enrol in, complete, and comply with the intervention [13,109]. The feasibility of the intervention was monitored with the research activity log and included assessment of: a) the proportion of persons who were screened and found eligible; b) the proportion of eligible participants who enrolled in the study; c) the dose of the intervention, defined as the number of home visits and case conferences during the six-month intervention; d) the level of fidelity to treatment (the extent to which the RNs and PSWs adhered to the components of the intervention); and e) the proportion of participants who withdrew from the study before completing the follow-up interviews. Reasons for non-consent or withdrawal were recorded, and comparisons of the characteristics of consenters vs. non-consenters and completers vs. non-completers were made.

### Acceptability of the intervention

Assessment of acceptability refers to the older home care clients’ and the study RNs’ and PSWs’ perceptions about the intervention’s appropriateness, benefits, and barriers and facilitators to implementation [109]. Perceptions of the intervention by study participants were measured using semi-structured interviews during the final one-year interview. Perceptions of the intervention by the study RNs and PSWs were measured using focus group interviews at six and 18 months following initiation of the intervention.

### Effects of the intervention

The *primary measure of effect* was the change in severity of depressive symptoms from baseline to six months as measured by the Centre for Epidemiological Studies in Depression (CES-D) score [110]. The CES-D consists of 20 items; the scores range from 0–60, with higher scores indicating greater severity. A cut-off score of 16 defines a clinically significant level of depressive symptoms, and a score of 21 or higher defines a moderate to severe level of depressive symptoms [110].

*Secondary measures of effect* included changes in the following variables from baseline to six months and one year: a) prevalence of clinically significant depressive symptoms using a cut-off score of ≥ 16 on CES-D, b) severity of anxiety measured by the Generalized Anxiety Disorder Screener (GAD-7) scale [111], c) prevalence of anxiety disorder using a cut-off score of ≥ 5 on GAD-7 [112], and d) HRQoL measured by the SF-12v2 health survey [113]. All tools have established reliability and validity.

The *costs of use of all types of health services* were determined using the Health and Social Services Utilization Inventory (HSSUI), which assesses costs from a societal perspective [114]. The HSSUI consists of questions about the respondent’s use of six categories of direct healthcare services: a) primary care, b) emergency department and specialists, c) hospital days, d) seven types of other health and social professionals, e) prescribed medications, and f) lab services. The six-month cost data were derived from the product of “quantity” data reported on the HSSUI and 2009–2012 “price” data obtained by our team for the HSSUI [114]. The costs of use of health services included the costs associated with delivery of the nurse-led intervention. Administrative data from the CCAC was used to determine the use of CCAC services.

### Sample size calculation

Sample size was calculated to detect a clinically important difference of 3.5 points in mean change from baseline to six months in the primary outcome measure, severity of depressive symptoms. Using a standard deviation of 9.0 as a conservative estimate, a sample size of 105 was estimated to be sufficient to address this primary outcome (two-tailed alpha = 0.05; beta = 0.20).

### Data analyses

Quantitative data were analysed using the Statistical Package for the Social Sciences (SPSS) version 19.0 for Windows, using two-sided tests at the 0.05 level of significance. For all models, the results were expressed as effect (or odds ratio for binary outcomes), standard errors, corresponding two-sided 95% confidence intervals (CI), and associated p-values. Descriptive analyses of participants’ characteristics and feasibility of the intervention were expressed as a mean (standard deviation [SD]) or median (minimum-maximum) for continuous variables and count (percent) for categorical variables.

Changes in outcomes over time were examined using paired t-tests for continuous variables and Chi-square test (or Fisher’s exact test) for categorical variables. We used normality probability plots to assess normality and used non-parametric tests (Kruskal-Wallis and Wilcoxin Signed-Rank Test) if the normality assumption was seriously violated. Because cost data are often right skewed, non-parametric tests were used to evaluate differences in medians between two time periods. However, policy-makers and decision-makers are concerned with the total costs of treating all older home care clients (including those with low and high consumption of services). As a result, mean cost is recommended as the most appropriate measure to describe the cost of a program or service [115].

We also performed an adjusted analysis to examine the relation between the predictor variable (total number of home visits by the study RN and PSW) and the primary outcome variable (six-month CES-D score) using backward regression analysis. The predictor variable and potential confounding variables representing known risk factors for depression were included in the initial models. We assessed multicollinearity by investigating associations among the confounding variables. Variables considered collinear were excluded from the analysis [116]. The possibility of co-interventions, such as mental health-related counselling and support programs, was also monitored.

Content analysis of the older home care clients’ responses to the open-ended questions was used to analyse the qualitative data. Participant answers were sorted into categories and themes. Descriptive analysis was performed for the frequency of the answers for each question. The RN and PSW focus group sessions were digitally recorded and transcribed verbatim. Qualitative data were managed using N-VIVO 9 software. Three of the investigators (MMR, CM, DF) systematically reviewed all transcripts and inductively generated a list of codes by hand describing themes. By comparing and contrasting the coded data, sub-themes, themes, interrelationships and patterns were revealed. Differences of opinion were discussed until agreement was reached.

## Results

### Feasibility of the intervention

#### Eligibility rate

**Figure 2:**
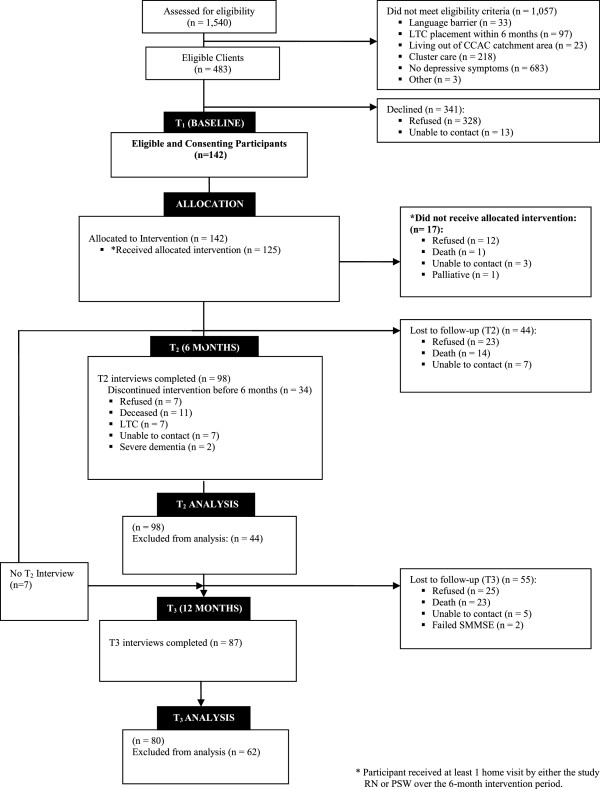
Study flow diagram.

Recruitment was conducted over a 13-month period from May 2010 to June 2011. Figure 2 provides a summary of the flow through the study. A total of 1,540 consecutive CCAC clients were screened for the study, and 483 (31%) screened positive for depressive symptoms and met all eligibility criteria. The most common reason for ineligibility was absence of depressive symptoms (65%).

#### Enrolment rate

In total, 142 (29%) of the 483 eligible home care clients consented and entered the study. Reasons for refusal to enrol in the study included unwillingness to change from their usual PSW to the study PSW (38%) and feeling overwhelmed or unwell (20%). Compared with consenters, more non-consenters answered “yes” to the screening question about losing interest or pleasure in doing things and were men. Fewer non-consenters had Parkinson’s disease.

#### Dose of the intervention

Of the 142 eligible and consenting older home care participants, 125 (88%) received at least one home visit by either the study RN or PSW during the six-month intervention period. Reasons for not receiving the intervention are shown in Figure 2. Twelve consenters subsequently refused to participate, some giving reasons of feeling unwell (25%) or lack of interest in the study (17%). Thirty-four additional participants (24%) discontinued the intervention early (reasons shown in Figure 2).

The frequency and timing of the home visits and case conferences were tailored to individual client needs and the results of the RN and PSW team’s assessment. Participants received a mean of six home visits (SD 4) over the six-month study period, a mean of 2.7 home visits by the RN (SD 2.2) and 3.4 home visits by the PSW (SD 2.4). Participants were discussed a mean of two times at the case conferences. More than one-third of participants (38.7%) were not discussed at a case conference; 27.5% were discussed one or two times, 18.7% were discussed three or four times, and 15.4% were discussed five or more times.

The delivery rates of the other components of the intervention were 78% for depression education, 72% for social and behavioural activation, 67% for medication review and supporting antidepressant medication management, 48% for the Home Support Exercise Program, 42% for referral and linkage to health and social services (including primary care), 37% for problem-solving therapy, and 28% for development of an IP depression management plan. The delivery rates of the validated screening tools were 84% for GDS-15, 81% for GIP-28, 78% for SMMSE, 75% for GAD-7, 71% for the Confusion Assessment Method (CAM), 64% for SCREEN II, and 35% for the problem-solving test. These proportions are based on the 125 older home care participants who received at least one home visit by a study RN or PSW.

#### Attrition rate

Of the 142 enrolled participants, 98 (69%) successfully completed the six-month follow-up. A total of 44 participants were lost to follow-up, yielding an attrition rate of 31% at six months. Reasons for loss to follow-up are shown in Figure 2. Of the 142 enrolled participants, 87 (61%) successfully completed the one-year follow-up, including seven participants who had not completed the six-month follow-up (Figure 2). A total of 55 participants were lost to follow-up, yielding an attrition rate of 39% at one year. Reasons for loss to follow-up are shown in Figure 2. The six-month and one-year analyses were based on a sample of 80 participants, for whom complete baseline, six-month, and one-year follow-up data were available.

#### Comparison between drop-outs and completers

The baseline characteristics of older home care client participants who completed the whole study (n = 80) were compared to those of participants who did not provide complete baseline, six-month and one-year follow-up data (n = 62). Compared to completers, drop-outs had significantly higher rates of peripheral vascular disease and 11 or more chronic health conditions, lower mean scores on the SF-12 general health perception and energy/vitality subscales, and higher per-person costs of use of acute hospitalization. Drop-outs were less likely to report a history of depression and had lower per-person costs of use of physician specialists and Meals on Wheels. There was no difference between drop-outs and completers on any other baseline characteristic.

#### Baseline demographic profile and depression-related clinical characteristics

The study eligibility criterion of having depressive symptoms was fulfilled by all 142 participants: 113 (80%) reported a loss of interest or pleasure in doing things and 135 (95%) reported feeling down, depressed, or hopeless most of the day, more days than not. Most participants (75%) answered yes to both questions. Clinically significant depressive symptoms (≥ 16 on CES-D) were found in more than half (56%) of participants, and more than one third (38%) had moderate to severe depressive symptoms (≥ 21 on CES-D). About one half (44%) of participants were taking at least one antidepressant medication. Sixty-four participants (45%) had a history of depression for an average of 5.7 years (SD 13.4). Sixty participants (42%) screened positive for both depressive symptoms and anxiety (≥ 5 on GAD-7). One hundred and three participants (73%) had clinically significant depressive symptoms or were taking an antidepressant medication. Of that number, only 22% were adequately treated (taking an antidepressant medication and scoring < 16 on CES-D).

Participants had an average of nine risk factors for depression (SD 2.5), including three or more co-morbid health conditions (96%); limitations in activities of daily living related to physical health (88%); cardiovascular disease (84%); use of five or more prescription medications (80%); female gender (65%); age 80 years or over (59%); hospital admission in last six months (59%); recent stressful life event (56%); anxiety disorder (51%); chronic pain (51%); widowed, divorced, or separated (48%); history of depression (45%); antidepressant medication use (44%); living alone (31%); and cognitive impairment (31%).

Participants were mostly women (65%), living with spouse or family (69%), with an average age of 82 years. A similar proportion was married (49%) or widowed/divorced (48%). Most (73%) had annual incomes of less than $40,000. Although 31% lived alone, almost all reported receiving some form of support from a family caregiver or a friend. Most were fairly ill: 59% reported one or more hospital admissions in the last six months, 79% suffered from six or more chronic health conditions, 31% were cognitively impaired, and 80% were taking five or more prescription medications daily. More than half (56%) of the participants reported a recent stressful life event, including personal illness or injury (48%), hospitalization (10%), change in residence (10%), lack of family support (10%), death of close family member or friend (8.1%), death of spouse (7.3%), change in health of family member (4.8%), and change in financial state (1.8%). Older adult participants reported SF-12 subscale scores at baseline that were significantly lower than published norms for the Canadian population, indicating poor HRQoL [117].

### Acceptability of the intervention

#### Perceptions of the intervention by older adult study participants

Overall, older home care client participants viewed the IP nurse-led mental health promotion intervention as highly acceptable. In addition to treating depression, the nurse-led intervention was credited with other benefits, such as instilling hope, and increasing clients’ mobility, function, confidence levels, and ability to live independently. Older adult participants often stated that the RN and PSW were caring and available to spend time with them, providing emotional support, reassurance, and encouragement. This was perceived as a key factor in treating their depression and enhancing their quality of life. The clients also highlighted the RN as being effective in facilitating timely access to various services and supports. They felt the RN was competent, providing timely assessment and management of their health issues. Participants valued the assistance they received from the PSW in completing basic activities of daily living. The clients acknowledged that the RN improved their knowledge of depression assessment and management and helped in managing their medications. Overall, the participants acknowledged the RNs and PSWs as being great resources and committed to addressing their depression issues.

Few negative aspects of the intervention were identified by clients. These included the clients experiencing stress or embarrassment because of the stigma of mental illness. Participants also indicated that there was a need for more home visits, more support for family caregivers, and improved communication among home care providers, clients, and family caregivers.

#### Perceptions of the intervention by the home care providers

All providers were women, with an average age of 48 years and an average of 13 years experience in their current discipline. RNs and PSWs participated in separate focus groups in order to evaluate the feasibility and acceptability of the IP nurse-led intervention. They communicated three main themes: the benefits of the intervention; barriers to effective depression management and successful implementation of the intervention; and recommendations for improving the sustainability of the IP nurse-led approach.

#### Benefits of the IP nurse-led intervention

Overall, the home care providers viewed the intervention as highly acceptable and were able to describe a full range of benefits for clients, providers, and the organization.

##### For clients

In addition to improving the recognition and management of depression, the nurse-led intervention was credited with other benefits, such as increasing clients’ physical and pleasurable activities, social support, quality of life, and ability to manage other chronic health conditions. Also, the RNs acknowledged that the intervention, through education, improved clients’ knowledge of the symptoms of depression, available treatments, and community resources available for depression care. Providers indicated that family caregivers valued the recognition and support they received for their caregiving role and responsibilities.

##### For home care providers

Both RNs and PSWs acknowledged the importance of mental health promotion and their role in the prevention and management of depression among community-living older adults. The most frequently cited benefit was that the nurse-led intervention improved IP communication, collaboration, and teamwork, as well as their own knowledge about depression assessment and management. The providers acknowledged the importance of the partnership between the RN and the PSW, in that the collaboration allowed each provider to do her own job better. Regular contact with study clients was cited as essential in establishing a confiding nurse-patient relationship, which was considered itself to be therapeutic.

##### For organizations

Providers indicated that implementing the nurse-led intervention positioned their agency as a leader in providing community-based depression care, which is not typically the focus of home care services. Providers also acknowledged the positive effect of the intervention on building capacity in depression care within their own agencies and fostering the development of partnerships among community organizations involved in providing mental health services to older adults. Implementation of the intervention was seen as highly compatible with the existing priorities of the home care program.

#### Barriers to depression management and implementation of the nurse-led intervention

The RNs and PSWs identified several barriers to successful implementation of the nurse-led intervention. Issues that need to be addressed include improving provider knowledge about depression care and the management of older adults with complex chronic conditions; addressing the stigma of mental illness; improving communication among the RN, PSW, CCAC case manager, and PCP; scheduling regular case conferences; improving provider knowledge and use of validated screening tools; establishing the role of home care nurses in the area of mental health promotion; and reducing the number of screening tools. The providers also identified several factors that impeded the effectiveness of the intervention, including restrictive eligibility criteria; limited funding; heavy workloads and limited time; competing team member priorities; difficulties communicating with the PCP; and lack of continuity and coordination of care between home care, acute care, and primary care settings.

#### Recommendations for improving the sustainability of the nurse-led intervention

Home care providers made several recommendations to improve the sustainability of the nurse-led intervention.

1) Make depression awareness, assessment, and treatment a priority within home care.

2) Expand the eligibility criteria for the intervention to include younger seniors with depressive symptoms.

3) Enhance education and training of home care providers in the prevention, recognition, and management of depression.

4) Simplify the intervention by creating a depression care pathway that helps providers determine the nature and severity of the client’s depression and outlines the best course of treatment based on the client’s specific needs and preferences. Provide guidelines and resources to support the implementation of the care path in routine practice.

5) Provide education to enhance IP collaboration and address barriers to communication and collaboration between home care providers, and between home care and primary healthcare providers.

### Effect of the intervention on the prevalence and severity of depressive symptoms

**Figure 3:**
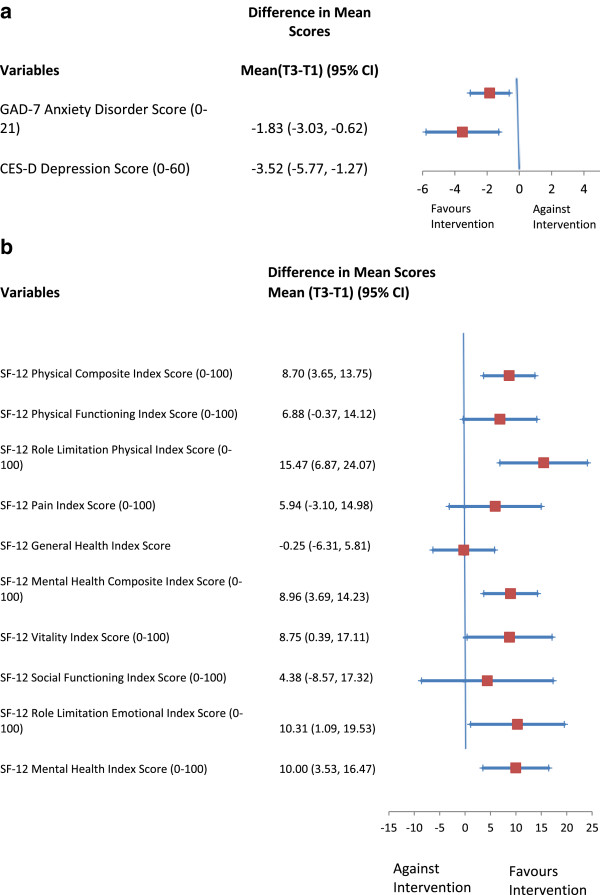
**Mean difference in older home care clients’ depressive symptoms, anxiety, and health-related quality of life from baseline to one-year.** Follow-Up (n=80): **(a)** Mean Difference in Older Home Care Clients’ Depressive Symptoms and Anxiety from Baseline to One-Year Follow-Up (n=80). **(b)** Mean Difference in Older Home Care Clients’ Health-Related Quality of Life from Baseline to One-Year Follow-Up (n=80).

At baseline, the mean CES-D score was 17.7 in the 80 participants that completed the study. The results of the paired t-test showed a statistically significant and clinically important decrease in the CES-D score from baseline (T1) to six-month follow-up (T2) (difference in mean scores, T2 vs. T1 -3.22, 95% CI: −5.35to −1.08, p = 0.004, followed by an additional reduction in the CES-D scores at one-year follow-up (T3) (difference in mean scores, T3 vs. T1 -3.52, 95% CI: −5.77 to −1.27, p = 0.003) (Table 4 and Figure 3(a)). These differences translated into a 62% reduction over the study period in the proportion of clients with clinically significant depressive symptoms (≥ 16 on CES-D), from 55% at baseline to 21% at one year.

**Table 4:** Changes in depression, anxiety and HRQoL over the study period (n = 80)

		**Time 1**^**a**^		**Time 2**^**b**^				**Time 3**^**c**^			
	**n**	**M**	**SD**	**M**	**SD**	**p-value (T2-T1)**	**Difference in mean scores (T2-T1) (95% CI)**	**M**	**SD**	**p-value (T3-T1)**	**Difference in mean scores (T3-T1) (95% CI)**
CES-D Depression Score (0–60)	79	17.71	10.65	14.49	10.57	0.004	−3.22 (−5.35, −1.08)	14.19	10.94	0.003	−3.52 (−5.77, −1.27)
GAD-7 Anxiety Disorder Score (0–21)	80	5.98	5.43	5.03	5.75	0.125	−0.95 (−2.17, 0.27)	4.15	5.14	0.003	−1.83 (−3.03, −0.62)
SF-12 Physical Composite Index Score (0–100)	80	32.85	21.09	42.38	25.09	0.001	9.53 (3.85, 15.20)	41.55	23.49	0.001	8.70 (3.65, 13.75)
SF-12 Physical Functioning Index Score (0–100)	80	13.13	24.83	21.25	30.06	0.025	8.13 (1.06, 15.19)	20.00	30.66	0.063	6.88 (−0.37, 14.12)
SF-12 Role Limitation Physical Index Score (0–100)	80	25.94	26.59	42.34	38.44	0.001	16.41 (7.16, 25.65)	41.41	36.11	0.001	15.47 (6.87, 24.07)
SF-12 Pain Index Score (0–100)	80	53.75	39.60	61.88	37.73	0.031	8.13 (0.78, 15.47)	59.69	38.87	0.195	5.94 (−3.10, 14.98)
SF-12 General Health Index Score (0–100)	80	45.50	33.99	44.06	31.18	0.701	−1.44 (−8.87, 6.00)	45.25	27.88	0.935	−0.25 (−6.31, 5.81)
SF-12 Mental Health Composite Index Score (0–100)	80	54.48	19.91	60.99	23.13	0.013	6.51 (1.38, 11.64)	63.44	23.27	0.001	8.96 (3.69, 14.23)
SF-12 Vitality Index Score (0–100)	80	24.06	26.82	28.13	30.39	0.360	4.06 (−4.72, 12.85)	32.81	31.72	0.040	8.75 (0.39, 17.11)
SF-12 Social Functioning Index Score (0–100)	80	49.69	40.66	51.25	40.35	0.771	1.56 (−9.11, 12.23)	54.06	42.55	0.503	4.38 (−8.57, 17.32)
Role Limitation Emotional Index Score (0–100)	80	69.53	30.48	78.59	32.81	0.040	9.06 (0.43, 17.69)	79.84	33.13	0.029	10.31 (1.09, 19.53)
SF-12 Mental Health Index Score (0–100)	80	57.03	24.67	64.69	29.28	0.026	7.66 (0.95, 14.36)	67.03	29.85	0.003	10.00 (3.53, 16.47)

^a^Time 1 (T1): Baseline.

^b^Time 2 (T2): Six-month follow-up.

^c^Time 3 (T3): One-year follow-up.

M = Mean SD = Standard Deviation.

Backward regression was used to conduct an adjusted analysis of the relation between the dose of the intervention (total number of home visits by the study RN and PSW) and the six-month CES-D score. Three baseline variables, CES-D score (β = 0.58), presence of an anxiety disorder (≥ 5 on GAD-7) (β = 3.93), and recent stressful life event (β = 3.28), explained 41% of the variance in the six-month CES-D score (p = 0.01). Hence, the severity of depressive symptoms at six-month follow-up was not related to the total number of home visits. Depressive symptoms were most strongly related to the presence of an anxiety disorder and a recent stressful life event, and less strongly related to the baseline CES-D score.

### Effect of the intervention on the prevalence and severity of anxiety

At baseline, the mean GAD-7 score was 5.98 in the 80 participants that completed the study. The results of the paired t-test showed no significant difference in the GAD-7 score from baseline to six months (difference in mean scores, T2 vs T1 -0.95, 95% CI: −2.17 to 0.27, p = 0.13, but there was a significant reduction in the GAD-7 score at one year (difference in mean scores, T3 vs T1 -1.83, 95% CI: −3.03 to −0.62, p = 0.003) (Table 4 and Figure 3(a)). These differences translated into a 49% reduction over the study period in the proportion of clients with an anxiety disorder (≥ 5 on GAD-7), from 51% at baseline to 26% at one year (p = 0.002).

### Effect of the intervention on health-related quality of life

At baseline, older adult participants had poorer HRQoL than the general Canadian population. The results of the paired t-tests showed that participants improved in all SF-12 dimensions of HRQoL over the study period, with statistically significant (at one or both follow-up times) and clinically important improvements in eight dimensions: physical composite score, physical functioning score, role functioning related to physical health score, pain index score, mental health composite score, vitality index score, mental health functioning score, and role functioning related to emotional health score (Table 4 and Figure 3(b)). A difference of five points for a dimension of the SF-12 is considered clinically and socially important [118].

### Effect of the intervention on depression management knowledge and practices

Almost half of older adult participants reported taking an antidepressant medication at baseline. The results of the Chi-square test indicated that, from baseline to six-month follow-up, the proportion of older home care clients taking prescription antidepressants increased, from 44% to 51% (p < 0.001). However, there was no difference in the proportion of clients using antidepressant medications from baseline to one-year follow-up. There was also no difference in participants’ self-reported use of specialized mental health services from baseline to six months or one year. No significant correlation was found between the number of antidepressant medications and the CES-D score at six months.

Positive changes in the clinical practice behaviours of study RNs and PSWs occurred over the intervention period. There was also a significant increase in the study RNs’ self-reported knowledge and confidence in caring for community-living older adults with depressive symptoms(e.g., assessing depressive symptoms using validated tools; screening for risk factors for depressive symptoms; providing depression education; providing social and behavioural activation; conducting a medication review; providing a structured in-home exercise program; developing IP care plans related to depression management; and documenting the effectiveness of depression interventions). There was also a moderate increase in the study PSWs’ self-reported knowledge and confidence in caring for community-living older adults with depressive symptoms over the intervention period. This self-report data was quantitative in nature and was part of the sociodemographic questionnaire administered to the RNs and PSWs prior to the focus group.

### Effect of the intervention on the costs of use of health services

Costs for the intervention, based on the older adults who participated in the intervention, were $189 for a nursing home visit, $78 for a PSW home visit, and $230 for a case conference. The mean total cost of providing the six-month nurse-led intervention was $497 per participant. The results of the Wilcoxin Signed-Rank test, based on the 80 participants that completed the study, showed no significant difference in the total mean per-person costs of use of all types of health services (including the nurse-led program costs) from baseline to six months (difference -$3,101, 95% CI: −$11,545 to $5,343; p = 0.47), or from baseline to one year (difference -$6,130, 95% CI: −$14,709 to $2,449; p = 0.16).

However, there were significant differences over the study period in the mean per-person costs of use of specific types of health services, including reductions in the costs of use of acute hospital days from baseline to six months (difference -$8,832, p = 0.03) and one year (difference -$9,126, p = 0.03). This difference was due to a 56% reduction in the number of participants with one or more hospital admissions over the study period, from 55% (44/80) at baseline to 24% (19/80) at one year (p = 0.44). There were also significant reductions from baseline in the mean per-person costs of use of ambulance services (difference -$105 at six months, p = 0.002 and -$138 at one year, p < 0.001) and emergency room visits (difference -$89 at six months, p = 0.006 and -$104 at one year, p = 0.002).

These reductions in costs were offset by increases, from baseline to one year, in the costs of use of other health providers (e.g., dentist, optometrist, massage therapy) (difference $1,328, p = 0.02), and long-term care (difference $1,011, p = 0.02). This difference in costs of long-term care was due to 14% of older adults being admitted to long-term care over the study period. There was no significant difference in the costs of use of other types of health services.

## Discussion

### Feasibility of the intervention

The feasibility of the intervention relates to the degree to which the participants enrol in, complete, and comply with the intervention [46]. Despite concerted efforts, the 29% enrolment rate in this study was lower than the 45-64% rates reported in other similar studies [3,86,90,92], a difference that may be related to the nature of the intervention or the target population. Our lower enrolment rate could also be related to older adults’ reluctance to report depressive symptoms. In general, the reasons given for non-participation were not related to the nature of the intervention. The challenges of recruiting older adults with depression are well documented [119,120]. To address recruitment barriers, we used clear but simple communication, gave the client the time needed to decide, had a clear protocol for contacting potential participants and flexible scheduling, and educated family caregivers about the study. Our efforts confirm that, although recruitment was labour-intensive and difficult, it was achievable in this client group.

We had attrition rates of 31% at six months and 39% at one year, which are comparable to those reported in other similar studies [86]. Client characteristics, organizational barriers, and client preferences contributed to attrition in our study. First, 16% of participants died over the course of the study, while 18% discontinued the study because of health problems or lack of interest, and the remainder were difficult to contact. Again, the reasons given for withdrawal were not related to the nature of the intervention.

Attrition has been recognized as a factor that threatens internal validity and reduces statistical power in a study. To minimize attrition, the study coordinator used a participant-tracking plan and the interviewers built rapport and trust with the participants and maintained between-assessment contact [121,122]. Participants were also compensated for their time ($15 for the baseline interview and $10 for the six-month interview). Case manager recruiters and interviewers met monthly with the principal investigator to clarify recruitment and data collection procedures, identify problems, and make suggestions for improvement. Attrition may contribute to self-selection bias when the characteristics of individuals who withdraw from the study differ from those of individuals who complete the study [123]. In our study, drop-outs were a somewhat lower-functioning group than those who were retained in the study.

Compliance with the intervention was operationalized as: (1) the dose of the intervention, defined as the number of home visits and case conferences, and (2) the level of fidelity to treatment (i.e., the extent to which the RNs and PSWs adhered to the components of the intervention). About 88% of older adult participants received at least one home visit by either the study RN or PSW during the six-month intervention period. Of that number, 73% completed the six-month intervention and 27% withdrew. This completion rate is comparable to those reported in other similar studies [3,86,90,92]. The delivery rate of the components of the intervention ranged from 28% for development of an IP depression management plan to 78% for depression education. In addition, 71 to 84% of participants were screened for depressive symptoms, cognitive impairment, anxiety, and delirium. Variations in the delivery rate of these components may reflect tailoring of the intervention to individual clients, providers, and settings. The smaller time commitment to the development of an IP depression management plan could have been related to the smaller number of RNs compared to PSWs on the team. Indeed, the RNs reported heavy workloads and limited time as barriers to implementation of the intervention. Future research exploring the composition and distinct roles of the members of the nurse-led teams, including optimal RN:PSW ratios is warranted.

Several strategies were implemented to monitor and enhance fidelity of intervention implementation. These included: (1) monthly *audits* of the study documentation to assess fidelity, (2) monthly *outreach visits* with the intervention providers, and (3) scheduled *reminders and updates*[108]. These strategies proved to be effective in identifying problems related to implementation, clarifying the intervention protocol, and developing suggestions for improving fidelity of intervention implementation.

### Acceptability of the intervention

Acceptability was defined as the older home care client and study RNs’ and PSWs’ perception of the intervention’s appropriateness, benefits, and convenience of implementation [109]. Overall, the participants viewed the nurse-led intervention as highly acceptable and were able to describe a full range of benefits. Perceived benefits from the client perspective centred on the personal attributes of the providers (caring and competent) and positive results (decreasing depression, improving function, instilling hope, increasing confidence, increasing knowledge of depression, and facilitating access to other services and supports). Older adult participants also indicated that caring, emotional support, reassurance, and encouragement were key factors in the treatment of their depression. This was supported by the qualitative feedback from the providers. These findings suggest the need to include a relational measure of some kind in future studies to quantify the impact of this important aspect of the intervention on the outcomes. Family caregivers indicated that they also valued the recognition and support they received as a result of the intervention. This finding suggests that future IP nurse-led DCM interventions should include a family satisfaction measure of some kind to capture the impact of the intervention on family caregivers.

Providers highlighted the benefits of: (1) regular in-home visits, (2) IP collaboration and teamwork, (3) increased knowledge of depression assessment and management, (4) the RN working collaboratively with the PSW, (5) improved communication and collaboration among home care, primary healthcare, and specialized mental health professionals and services, (6) improved recognition and management of depression, and (7) access to timely primary care and follow-up management.

Key barriers to implementing the nurse-led intervention included: (1) lack of home care provider knowledge and skills in depression assessment and management in older adults with complex chronic conditions, (2) addressing the stigma of mental illness, (3) heavy workloads and limited time, (4) communication barriers among the RN, PSW, home care CM, and PCP, and (5) limited access to personal support and other home care services. Recommended solutions to improve the sustainability of this approach to care delivery included: (1) expanding eligibility criteria, (2) providing additional staff education about depression care management, (3) improving communication among home care providers, and (4) developing a depression care pathway. These findings are similar to those of recent research on implementing evidence-based mental health programs in community settings [89]. Our study suggests that future IP nurse-led DCM interventions should incorporate these strategies to improve the effective translation of this evidence-based approach to care.

### Effects of the intervention

This study provides initial evidence for the feasibility, acceptability, and sustained effects of the intervention in improving client outcomes, reducing use of expensive health services, and improving clinical practice behaviours of home care providers. Our results extend those in the literature and current translation of evidence-based depression care in several ways.

First, the IP nurse-led mental health promotion intervention proved to be feasible and effective in reaching our target group - older home care recipients with depressive symptoms. The baseline rate of clinically significant depressive symptoms of 56% in the present sample greatly exceeds the 8.5-47% rates reported for representative samples of older home care recipients [1,4,8,25,61,92]. Our study deliberately recruited only seniors who had an increased risk for depressive symptoms; in fact, their rate of depressive symptoms was closer to the rate reported among institutionalized older adults [124]. A key issue is not simply the high prevalence of depressive symptoms, but rather the combination of depressive symptoms and high rates of co-morbidities. Almost all the older adults in our study had multiple (3 or more) chronic conditions and 68% had six or more. This situation is cause for concern, given that depression in the context of multiple chronic conditions is associated with increased medical symptom burden, functional impairment, and poor adherence to treatment, increasing the probability of adverse health outcomes and increased healthcare utilization and costs [125]. Overall, these findings underscore the important role of home care in the screening, early identification, and management of depression in this vulnerable population.

Of the 142 eligible consenting older home care clients, 103 (73%) had clinically significant depressive symptoms or were taking an antidepressant medication. Of that number, only 22% were adequately treated, a rate consistent with the 20-30% rates observed in other studies [1,8,12]. These findings are noteworthy, given that untreated or under-treated depression is associated with greater morbidity and dependency, functional decline, diminished HRQoL, pain [14], poor adherence to medical treatment, increased demands on family caregivers, premature nursing home admissions [16], increased use of healthcare services [2,3,9,15-18], and increased risk of premature death from suicide and other medical conditions [19].

A key strength of our study was that the two-step screening and recruitment process identified many older adults who would not normally have received any treatment for their depression. This finding highlights the importance of incorporating depression screening, using a validated screening tool, into routine clinical practice for older adults requiring PSS. Based on the results of this study, screening should include evaluation of risk factors for depression, particularly anxiety, stressful life events, and a history of depression.

Second, the results of this study add to the growing evidence for the effectiveness of an IP nurse-led depression care management intervention for community-living older adults in reducing depressive symptoms [41,66,69,72,76,79,81-84,86] and improving HRQoL [41,42,70,76,79]. As we hypothesized, the IP nurse-led mental health promotion intervention was effective in reducing depressive symptoms at the six-month follow-up, with a small additional improvement six months after the intervention. The 20% (3.5 point) reduction in the mean CES-D depressive symptom score at one year is comparable to that reported in other DCM trials involving community-living older adults [41,42,66,69,72,76,79,81-85],[87,91,92] and is clinically meaningful [110]. This difference in the CES-D score translated into an impressive 62% reduction over the study period in the proportion of clients with clinically significant depressive symptoms.

Our study enrolled older adults with any level of depression severity, cognitive impairment, and other comorbid health conditions, including people who are often excluded from community-based studies. Almost one-third (31%) of the sample had dementia. Thus, this study makes an important contribution by providing knowledge of the effectiveness of a nurse-led mental health promotion intervention among a more vulnerable group of older home care clients. The use of less restrictive selection criteria increased the heterogeneity of the sample, reflecting the variability in older home care recipients seen in everyday practice. It also enhanced the generalizability and clinical applicability of the research findings to include older adults at risk of, suffering from, or recovering from depression [46,126].

An important finding of this study was that it provides preliminary evidence for the effectiveness of the intervention among older home care clients with dementia. This finding is particularly noteworthy given that depression in clients with dementia frequently remains undiagnosed or the depression is considered to be an inevitable and untreatable consequence of dementia. Our findings are consistent with those of previous studies that have shown that dementia in clients with depression does respond to treatment, and appropriate therapy can improve the well-being of these patients [127]. These findings suggest that future IP nurse-led DCM interventions should target older home care clients with dementia.

 A novel finding of this study was the long-term maintenance effect of the intervention in reducing depressive symptoms. This result is particularly meaningful, given the chronic and recurrent nature of depressive symptoms in this population. Most studies on the effectiveness of DCM interventions have analysed only the immediate effects of the intervention. Our findings suggest that clinical benefits continue to accrue well beyond the intervention period. Other studies have reported similar positive long-term effects on depressive symptoms [41,69] but they involved a nurse working in collaboration with an IP team, not leading an IP DCM strategy.

Another important finding of this study was the identification of three variables at baseline that predicted the severity of depressive symptoms at the six-month follow-up: anxiety, recent stressful life event, and history of depression. Little is known about the mechanism through which existing DCM interventions improve depression outcomes. Our study suggests that future IP nurse-led DCM interventions should give special attention to these factors as means of enhancing the effects of the intervention in reducing depressive symptoms. Our findings also suggest that limited home care resources may be used more effectively if targeted toward older adults with these characteristics.

Third, as expected, the intervention that reduced depressive symptoms also produced significant improvements in HRQoL. Given the lower level of HRQoL in older adult participants at baseline, this is a clinically important gain. Our findings are consistent with those of previous studies [41,42,70,76,79]; however, a novel aspect of our study was that the intervention effects on HRQoL were sustained six months after the intervention period. The association between depressive symptoms and HRQoL is well documented in the literature [70].

Fourth, a unique aspect of our study is that it included anxiety as an outcome, which is especially relevant in light of the high rate of co-morbidity between depression and anxiety [84], 42% in our sample, and the finding that anxiety at baseline was a risk factor for depressive symptoms. The intervention resulted in a 49% reduction over the study period in the proportion of clients with anxiety. It might be beneficial for future interventions to specifically target anxiety as a means of reducing depressive symptoms and enhancing the HRQoL of this vulnerable population.

Fifth, our study showed that these improvements in client outcomes were achieved at no additional cost to society as a whole, thus making the intervention highly desirable, given its clinical benefits. Previous studies that included an economic evaluation focused only on the use of hospital, emergency room visits, home care services, antidepressant medications, primary care, and specialty mental health services as measures of cost [73,74,86,93,95]. Our study is unique in that it measured use and costs of a full range of health services, from a societal perspective. Although our results showed no significant difference in the total mean costs of use of health services (including the nurse-led program costs), there were significant reductions in the costs of use of specific types of health services, such as acute hospitalization, ambulance services, and emergency room visits. Previous studies have also reported reductions in hospitalization with a collaborative IP DCM approach [73,74,86,95]. The $9,126 reduction in per-person costs of use of hospitalization by itself creates more than enough savings to pay for the intervention. In Canada, hospital costs constitute the largest component of healthcare expenditures for depression, at approximately $3.8 billion dollars per year [128].

Sixth, the study results support and extend the literature regarding best practice guidelines for the prevention and management of depression in older adults with depressive symptoms. Given the multifactorial nature of depression, older adults with multiple chronic conditions are best served by an IP team of professionals and non-professional PSWs with complementary skills to address the biopsychosocial determinants of depression. Our results support the need for a chronic disease management approach to depression that (1) involves an IP team; (2) targets individuals at risk [37]; (3) involves intensive RN case management and community navigation to facilitate timely access to services and supports [45,48]; (4) includes regular in-home visits by an RN and PSW; (5) encourages regular communication among home care providers, and (6) provides formal mechanisms for communication and collaboration between home care and primary healthcare providers and referral to specialized mental health services [48-50]. Moreover, our findings demonstrate the role and value of PSWs working in collaboration with an RN and other health professionals in enhancing client outcomes. Other studies have also shown the effectiveness of using trained PSWs to improve the health outcomes of older adults in community-based [7,106] and institutional settings [87,96].

Seventh, the intervention was effective in improving clinical practice behaviours of home care providers. This finding is noteworthy, because most interventions that have successfully improved depression detection have not led to better clinical practice [85]. The intervention had a significant effect on increasing antidepressant use among older home care participants at six months. Previous studies have also reported improvements in antidepressant use with a collaborative IP DCM approach [129,130]. It is possible that participants underreported use of mental health services, which was not increased during the study. Raina [131] found that older adults tend to over-report contact with primary care practitioners and under-report contact with other medical specialists. A future qualitative study is warranted to learn more about the barriers and facilitators to accessing specialized mental health services for older home care recipients.

Positive changes in many other clinical practice behaviours of the study RNs and PSWs occurred over the intervention period. There was also an increase in the study RNs’ self-reported knowledge and confidence in caring for community-living older adults with depressive symptoms. These findings suggest that the study home care providers were successfully trained to provide and deliver this evidence-based intervention for depression to older adults as part of their caseload. The importance of non-mental health nurses conducting this intervention is considerable, given that previous effectiveness studies of collaborative IP DCM approaches used trained mental health nurses or advanced practice nurses. The nurse-led mental health promotion intervention in this study was provided by existing home care staff.

Overall, our findings suggest that the IP nurse-led mental health promotion intervention is a promising model that has the potential for moving the field toward greater dissemination of evidence-based depression care into real-world practice settings. The academic and community agency partnerships worked together successfully on this research. The participating organizations demonstrated shared commitment for planning, implementation, and evaluation; shared vision and objectives; infrastructure support; stakeholder engagement and buy-in; and strong leadership support in the development of this nurse-led model. These are all essential factors that contributed to the development of a practical, transferable, and sustainable practice model in this population. The research built capacity in depression care and fostered collaborative partnerships across the geriatric mental healthcare delivery system that further enhanced the sustainability of the intervention.

### Limitations of the study

Several limitations to this study should be noted. First, the single study site may limit the generalizability of our findings. Second, there is no comparison group in the one-group pre-test post-test design. Third, despite concerted efforts, we were only able to enrol 29% of eligible clients; thus, our sample might not have been truly representative of the population at risk and sampling bias may have influenced the results. Fourth, it is possible that attrition resulted in self-selection bias, because the drop-outs were a somewhat lower-functioning group than those who were retained in the study. Fifth, clinical depression was not evaluated in this study; in future studies, it would be important to include a structured clinical interview to confirm a DSM-IV based diagnosis of major or minor depression versus depressive symptoms. Sixth, the use of a proxy respondent as a source of data for study participants with limitations in cognition, physical health, or language may have resulted in inaccuracies [132]. Seventh, the finding that there was no difference in the total per-person costs of use of health services from baseline to one-year follow-up may be because of an insufficient sample size and limited power to detect differences. Future trials with an economic evaluation are needed that have sufficient power to detect cost differences. A final limitation of the study design is that it is impossible to assess the specific contributions of each of the various elements of this complex IP nurse-led intervention. In future studies, it would be important to examine whether one or more of the components of the intervention are responsible for the effects or whether all components of the intervention are necessary ingredients. For example, future research is warranted to determine if those older adult participants referred by the RN to IP team members realized more benefits than those who were not referred to the IP team.

## Conclusions

With the rapid increase in the number of seniors living in the community, depression is becoming a serious problem that, without intervention, will place extensive burdens on healthcare resources. Home care has the potential to play a pivotal role in the prevention, early recognition, and management of depression as a means of enhancing the quality of life of older people with chronic conditions. The results of this study provide the first evidence of the feasibility, acceptability, and sustained effects of an IP nurse-led mental health promotion intervention in improving client outcomes, reducing use of expensive health services, and improving clinical practice behaviours of home care providers under real-world practice conditions. These findings are important, given the high prevalence of depression among older home care clients, and the low rate of recognition and treatment of depression in this high-risk population. The study findings underscore the role and value of nurses within the IP home care team in the management of depression and generate lessons learned that are relevant to other home care settings. Home care policy makers, agencies, and funders should consider an IP nurse-led strategy as an appropriate and effective approach to reducing depressive symptoms and improving the HRQoL of older adults with depressive symptoms. Future research should evaluate its efficacy using a randomized controlled trial design, in different settings, with a larger sample of older home care clients with depressive symptoms. Future research is also needed to identify strategies to improve enrolment and completion rates to ensure adequate sample size and to reach those most at risk.

## Abbreviations

PSS: Personal support services; DCM: Depression care management; RCT: Randomized controlled trial; IP: Interprofessional; RN: Registered nurse; PSW: Personal support worker; PCP: Primary care physician; HRQoL: Health-related quality of life; CCAC: Community care access centre; HNHB: Hamilton Niagara Haldimand Brant; CES-D: Centre for epidemiological studies in depression; SMMSE: Standardized mini-mental state examination; GAD-7: Generalized anxiety disorder screener-7; HSSUI: Health and social services utilization inventory; GDS-15: Geriatric depression scale-15; GIP: Behavioral rating scale for intramural psychogeriatric inpatients; CAM: Confusion assessment method; SCREEN II: Seniors in the community risk evaluation for eating and nutrition; PHQ-2: Patient health questionnaire-2; SD: Standard deviation; M: Mean; CI: Confidence interval.

## Competing interests

The authors declare that they have no competing interests.

## Authors’ contributions

All authors contributed to the design of this study. MMR, CM, DF, MG, GB, TP and BB contributed to the design and implementation of the nurse-led intervention. MMR wrote the first draft of this manuscript, and all authors contributed to the discussion and editing. All authors read and approved the final manuscript. LT supervised the statistical analyses, JSH supervised the economic analyses, and all authors had full access to the data.

## Pre-publication history

The pre-publication history for this paper can be accessed here:

http://www.biomedcentral.com/1471-2318/14/62/prepub
